# A Novel 65 nm Active-Inductor-Based VCO with Improved Q-Factor for 24 GHz Automotive Radar Applications

**DOI:** 10.3390/s22134701

**Published:** 2022-06-22

**Authors:** Prangyadarsini Behera, Abrar Siddique, Tahesin Samira Delwar, Manas Ranjan Biswal, Yeji Choi, Jee-Youl Ryu

**Affiliations:** 1 Department of Smart Robot Convergence and Application Engineering, Pukyong National University, Busan 48513, Korea; prangyadarsini.behera@gmail.com (P.B.); abrarkhokhar.iiui@gmail.com (A.S.); samira.fset@gmail.com (T.S.D.); mrbiswal13@gmail.com (M.R.B.); wl03845@gmail.com (Y.C.); 2 Department of Electrical and Computer Engineering, University of Waterloo, Waterloo, ON N2L 3G1, Canada

**Keywords:** low supply voltage, low phase noise, CMOS tunable active inductor (CTAI), voltage-controlled oscillator (VCO)

## Abstract

The inductor was primarily developed on a low-voltage CMOS tunable active inductor (CTAI) for radar applications. Technically, the factors to be considered for VCO design are power consumption, low silicon area, high frequency with reasonable phase noise, an immense quality (Q) factor, and a large frequency tuning range (FTR). We used CMOS tunable active inductor (TAI) topology relying on cascode methodology for 24 GHz frequency operation. The newly configured TAI adopts the additive capacitor $\left(C_{ad}\right)$ with the cascode approach, and in the subthreshold region, one of the transistors functions as the TAI. The study, simulations, and measurements were performed using 65nm CMOS technology. The assembled circuit yields a spectrum from 21.79 to 29.92 GHz output frequency that enables sustainable platforms for K-band and Ka-band operations. The proposed design of TAI demonstrates a maximum Q-factor of 6825, and desirable phase noise variations of −112.43 and −133.27 dBc/Hz at 1 and 10 MHz offset frequencies for the VCO, respectively. Further, it includes enhanced power consumption that varies from 12.61 to 23.12 mW and a noise figure (NF) of 3.28 dB for a 24 GHz radar application under a low supply voltage of 0.9 V.

## 1. Introduction

The progressively emerging prevalence and evolution of wireless communications and multi-standard radio frequency (RF) industries have brought together a number of networks with several hundred megahertz to gigahertz frequency channels, and this has significantly endorsed the compact transceivers in CMOS technologies in order to support power efficiency with the highest sharing of devices [1]. A unified transceiver chip continues to offer multimode, multi-band compatibility, which is indeed one of the salient features in designing the circuit for modern wireless communication systems. One of the vital building blocks in frequency translation is a fully optimized VCO used as a voltage-driven component in a phase-locked loop (PLL). The most challenging aspects for the implementation of a fully integrated VCO are chip dimensions, cost-effectiveness, low phase noise, wide frequency tuning range (FTR), high frequency, and low power consumption. In the high frequency transceiver, the PLL is the key design element and plays an important role in the overall receiver performance [2]. There has been a significant rise in ongoing research of millimeter wave PLLs that use less power and have more convenient architecture, thereby bringing the amount of insignificant content down to conventional standards [3,4]. Several kinds of PLLs are not appropriate for usage in the nodes that include a WSN. Several other designs that have lower phase noise and higher reliability standards, in addition to lowering the amount of power that is used, have been offered as solutions to this issue [5,6]. To utilize much of the wide frequency range, numerous studies have been performed to develop a VCO for the 24–30 GHz frequency band to improve the tuning range of the LC tank by compromising the quality factor. Despite the fact that a large frequency tuning range may be realized, the designs experience a significant increase in chip area and complications with their control mechanisms.

Due to their technological advantage in phase noise, LC-VCOs are commonly used to execute circuits, including spiral or passive inductors, and have been suggested in different approaches for tuning range improvement, but they suffer from complexity regarding chip area and low Q-factor. Whenever the PLL system is functioning at its frequency, it is essential to offer a better Q factor for the devices that make up the circuit. If the power consumption of WSN nodes can be lowered, their lifespans can be extended, the size of the batteries can be reduced, and the networks can become more compact [7,8]. Furthermore, if the system’s quality factor is inadequate, peak detection may be hampered, leading to results with significant uncertainty [9]. Passive inductors include specific attributes that drastically decrease the spectral efficiency, eliminate reliability, and maximize paradigm expenditures. To alleviate the issues, TAI has been introduced to enhance the tunable Q-factor, widen the FTRs of VCOs, and improve chip size by supplying low voltage in a 24 GHz transceiver design for radar applications. In this article, a novel cascade TAI topology is designed by evaluating the LC-CC oscillators to exhibit their performance characteristics. This study provides researchers with the freedom to refine TAI attributes and then to upgrade VCO functionalities when the fully-functional RF circuits are configured for the fully active RF circuits. In our effort, we describe a complete analysis for a TAI-VCO to perceive the realization in terms of chip dimension, phase noise, tuning range, Q-factor, resonance frequency ($\omega_{0}$), inductance, and power consumption by using low supply voltage. This study covers a frequency spectrum from 21.79 to 29.92 GHz, so as a result, both K-band and Ka-band are included. The K-band (IEEE) and Ka-band are defined as between the ranges of 18–27 GHz and 26.5–40 GHz, respectively.

### 1.1. Automotive Radar Spectra

**Figure 1:**
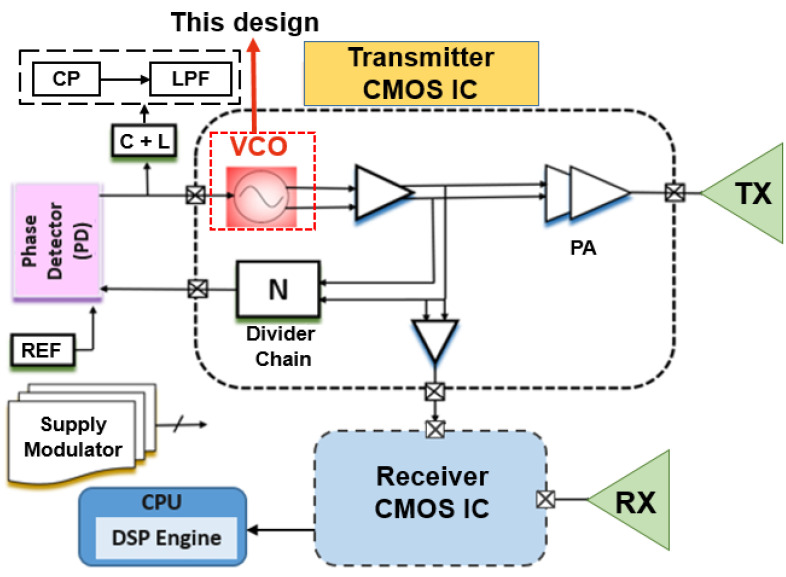
System-level architecture for 24 GHz radar communication.

**Figure 2:**
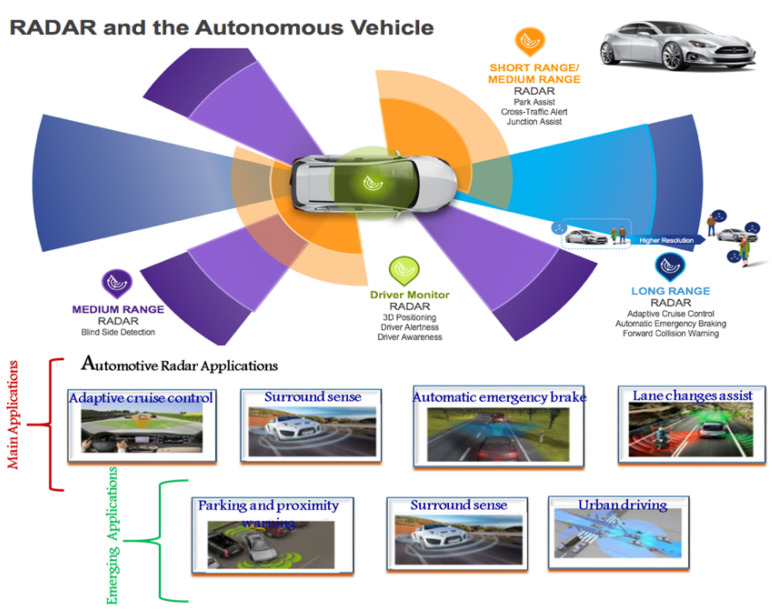
Automotive radar applications.

The radar transceiver for 24 GHz is mentioned in Figure 1. It shows the transmitter and receiver parts of the radar front-end, which consist of a phase detector (PD), low-pass filter (LPF), VCO, power amplifier (PA), divider chain (N), receiver CMOS integrated circuit (IC), and reference frequency as an input to the device. Transmitter and receiver CMOS IC, in combination with the CPU (DSP engine) and supply modulator, complete the system configuration, as shown in Figure 1. A substantial power factor and a low noise figure, as aforementioned, are essential for accurate range measuring. A power amplifier is integrated inside the transmitter to provide the necessary significant power [10]. Since discharge from the huge output swing of the power amplifier block might have a negative impact on the low noise amplifier, we designed the receiver and transmitter chips to be deployed independently. The supply modulator is responsible for providing $V_{dd}$ and bias. Figure 2 illustrates the application for automotive radar applications in the system architecture for the 24 GHz frequency spectrum. For use in vehicular automotive radar systems, a number of frequency bands have been designated. Figure 2 displays automotive radar functionalities and spectrum distributions that function in the 22–29 GHz band. SRRS is one of the applications shown in this figure. For ease, we shall refer to the aforementioned bands going forward as the 59.24 GHz/K-band. According to information provided by the FCC in the year 2002, SRRS devices may operate legally within a frequency spectrum spanning 22 to 29 GHz. The use of 24 GHz SRRS makes it possible to have features such as the identification of blind spots, aid with changing lanes and parking, and avoidance of collisions. Between 24.0 to 24.25 GHz is known as the the industrial, scientific, and medical 63 (ISM) band. This band is part of the 24 GHz spectrum. The Federal Communications Commission (FCC) announced in 2002 that SRRS was 64 legal throughout the frequency spectrum of 22 to 29 GHz [11].

### 1.2. Literature Review

The VCO is by far the most significant and essential component in signal conversion on and off transceiver networks. Even during the fabrication of VCOs, FTR, phase noise (PN), and millimeter wave (mmw) frequency development are the parameters that primarily concern process modification, having parasitic effects [12]. Researchers have explored several approaches in mmw technology to strengthen the VCO’s oscillation frequency and FTR, alongside considerably reducing phase noise levels [13,14].

The operational frequency of the VCO is governed by the device;s cutoff frequency. Meanwhile, the focus of such efforts is channeled towards the dimensional feature of the module to achieve a greater cutoff frequency. As the operating frequencies of the oscillator are restricted by the cutoff frequency of the device (transistors), most such work is directed at scaling down the device’s geometric profile in order to get a higher cutoff frequency [15,16,17,18,19,20].

Owing to the higher phase noise performance, LC-tank VCOs with spiral inductors and varactors are extensively adopted in radio frequency circuit implementations [21]. Theoretically, the VCO tuning range is determined by the maximum to minimum capacitance ratio of the varactor [22,23]. The tuning range of an LC-tank VCO is approximately restricted for a classical capacitance ratio in a typical CMOS process, rendering it unsuitable for wideband applications. Switched capacitors and switched inductors have been intended to enhance the tuning range of the LC-tank VCO [24]. Despite the fact that a large frequency tuning range can still be attained, the circuits undergo a significant rise in the chip space and the complication of the control mechanism used to employ devices. The technique of frequency tuning employing active inductors has indeed been constructed in order to mitigate the constraints placed on the tuning range [25].

The remainder of this work is outlined as follows. Section 2 includes the description of the CMOS tunable active inductor circuit design and explanations, whereas Section 3 defines the circuit design of the proposed TAI-VCO and characteristics of this novel mechanism. Section 4 clearly cover the simulation and measurement results, and post-simulation outcomes, respectively; the latter includes the system set-up and its layout in detail. Section 5 concludes with an informative summary.

### 1.3. Motivations

**Figure 3:**
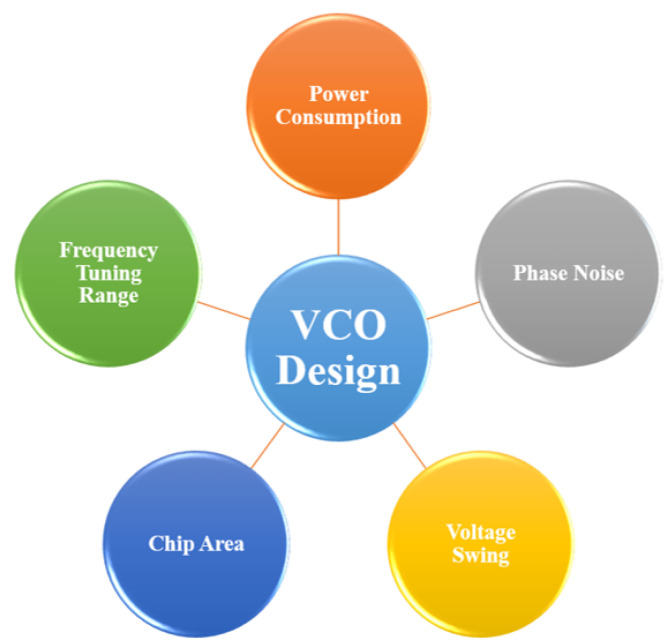
The VCO design trade-offs.

The increase in inductance that occurs from the configuration of a TAI-VCO is one of the primary factors to be taken into account, along with the quality factor. Despite this, LC-VCOs have limited tunable limits and very high silicon area consumption, both of which are results of spiral or passive inductors. A solution to this problem is available in the form of an active inductor, which may be used in place of a passive inductor. In addition, the active inductor is superior to the spiral inductor in terms of performance metrics, such as quality factor, silicon area usage, and tunabity. The feedback architecture of the gyrator-c topology is utilized by the active inductor as its primary method of operation. It is possible to get increases in phase noise performance and quality factor by incorporating a resistor into the feedback channel. This will result in an effective contribution to the enhancement of quality factor. There are descriptions of the traditional design trade-offs that are used in VCO design in Figure 3. In particular, since low power consumption is a primary issue, a low bias current is often one of the factors that is taken into account. However, there is a concern that this technique would enhance the parasitic effects, which will result in a worsening of the VCO phase noise. One on either hand, if you plan on having a low level of phase noise, you should think about having a better output voltage swing. There are a numerous approaches which can be used in order to accomplish this strategic goal. The designer can decide on a high bias current, which would lead to an increase in the amount of power that has to be used.

## 2. Advanced CMOS Tunable Active Inductor

**Figure 4:**
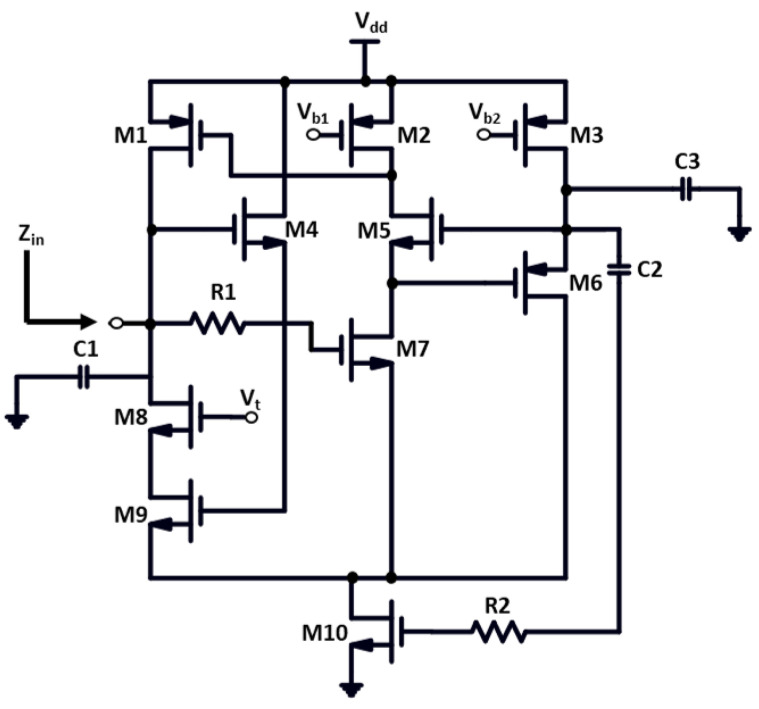
Proposed circuit design for an advanced tunable active inductor.

The persistence of parasite series resistance is often one of the greatest limitations when developing an active inductor. To mitigate parasitic series resistance, there are several methods, which include reducing output impedance and boosting the transconductance of the transconductor, by integrating the negative resistance or by using the simple cascode, or a regulated or multi-regulated cascade, in such a way as to lower the series resistance. Figure 4 illustrates the schematic view of the proposed tunable AI circuit, which embeds ten transistors (M1–M10), three capacitors (C1–C3) in which C1 and C3 act as additive capacitors, and two resistors (R1 and R2). The loop comprises transistors M5 and M6 that form feedback mechanisms built on gyrator theory, which implies that even in a very limited area relative to the spiral inductor, often 10s of nH of inductance are produced in several-gigahertz areas in this integrated circuit design. In the framework of this novel circuit architecture, the cascade structure of NMOS, the differential configuration of NMOS, and the usage of PMOS make the circuit less vulnerable to the noise level of the entire circuit and enhance the inductive bandwidth by ensuring the high-quality factor.

The NMOS mobility in CMOS technology is about four to five times higher than the mobility of PMOS. Consequently, the PMOS transistors aim to limit non-linearity and noise in the circuit, and with the N channel transistors, the high frequency performance of the active inductor is feasible, which significantly improves the operating frequency. The transistor $M_{7}$ is assembled beneath transistor $M_{5}$ in the circuit diagram, as shown in Figure 4. The supplemental gain stage has been assigned by the transistor $M_{6}$ in order to improve the cascode’s impact even further. The transistors $M_{1}$, $M_{2}$, and $M_{3}$ are the source supply terminals. The bias voltages $V_{b1}$ and $V_{b2}$ are linked to the source terminals of transistors $M_{1}$ and $M_{2}$, respectively. The voltage $V_{t}$ is responsible for monitoring the equivalent inductance of the active inductor and is coupled to the transistor $M_{8}$, which is stacked on top of the transistor $M_{9}$. $R_{2}$ and $C_{2}$ are linked to the transistor $M_{10}$, which is connected to the ground as a substitute for the tail current source; $M_{10}$ is being used to maintain the resistivity. As an outcome, the resistor $R_{1}$ has been incorporated in this research, in conjunction with $M_{7}$ and $M_{4}$, in addition to improving the inductance and Q-factor of this active inductor significantly. The $R_{1}$ resistor improves the impedance by generating extra inductive resistance that is driven towards the transistor $M_{7}$.

**Figure 5:**
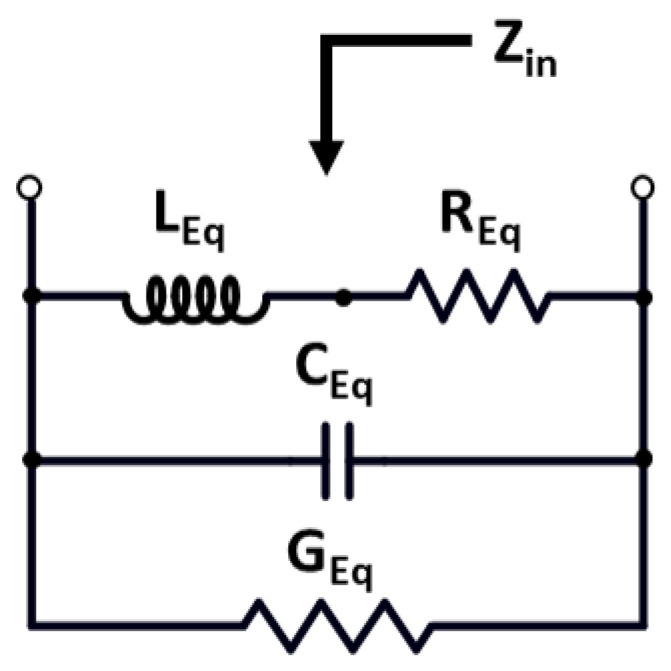
Equivalent circuit diagram of the proposed TAI.

The equivalent RLC circuit design is shown in Figure 5. Its effect is measured by the parameters input impedance $Z_{in}$, equivalent parasitic conductance $G_{Eq}$, equivalent parasitic capacitance $C_{Eq}$, equivalent parasitic resistance $R_{Eq}$, and equivalent inductance $L_{Eq}$.

**Figure 6:**
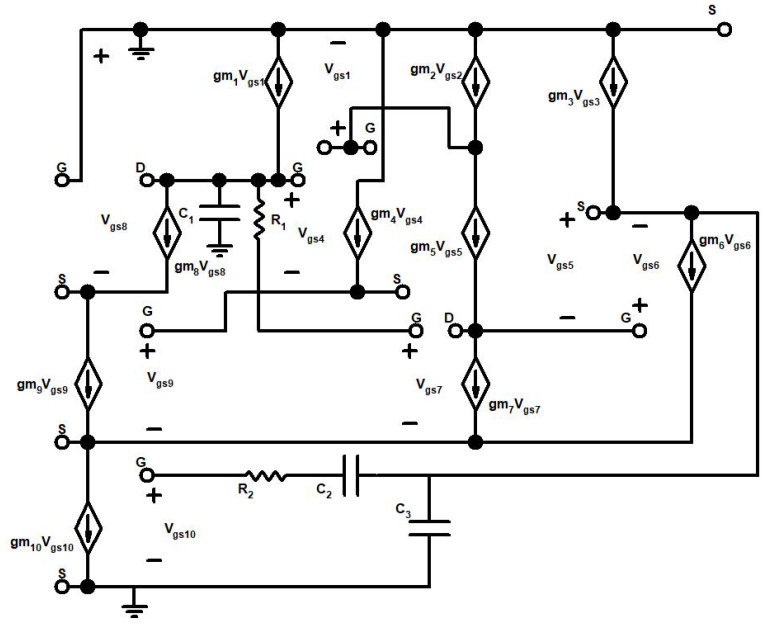
Small-signal analysis of the novel tunable AI.

Table 1 provides the detailed information for a comparison study of the passive and active inductors. The performance parameters are described for spiral inductors and for simulated active inductors in this table. Figure 6 depicts the representation of a small-signal analysis of the cascode tunable active inductor model stated in Figure 4. The function of the VCO for a wide tuning range is dependent on the tunable active inductor’s configuration. Furthermore, the behavior of the TAI is characterized by a small-signal analysis. The analysis outlines the optimized equivalent small-signal circuit of the active inductor M1–M10. For the analysis of TAI, gate-drain capacitance $C_{gd}$ being lesser than gate-source capacitance $C_{gs}$, taken as an assumption for the evaluation of system variables, is described by the following equations.

$Z_{in}$ can be written as

(1) $$Z_{in}=\frac{1}{Y_{in}}$$

The input admittance $Y_{in}$ can be expressed as

(2) $$\begin{array}{cc} Y_{in}=sC_{gs7} & +sCgs9+g_{ds7}+\frac{gm_{6}*gm_{7}}{sC_{gs1}(1+s\left(\frac{C_{gs5}}{gm_{5}}\right)+s^{2}(\frac{C_{gs5}}{gm_{5}})*\left(\frac{C_{gs6}}{gm_{6}}\right))}\\ & +\frac{\left(gm_{7}+sC_{gs7}\right)\left(gm_{9}+g_{ds9}+sC_{1}\right)}{\left(g_{ds9}+sC_{1}\right)\left(1+sC_{gs7}R_{1}\right)+sC_{gs7}}+\frac{gm_{7}}{1+s(\frac{C_{gs5}}{gm_{5}})+s^{2}((\frac{C_{gs5}}{gm_{5}})*\left(\frac{C_{gs6}}{gm_{6}}\right))} \end{array}$$

(3) $$G_{Eq}=g_{ds7}+\frac{gm_{7}}{1-\omega^{2}\left(\frac{C_{gs6}*C_{gs5}}{gm_{5}*gm_{6}}\right)}+\frac{C_{1}*gm_{7}+C_{gs7}\left(gm_{9}+g_{ds9}\right)}{C_{1}+C_{gs7}\left(1+g_{ds9}R_{1}\right)}+\frac{C_{gs9}*g_{d1}-\omega^{2}C_{1}C_{gs9}C_{gs7}R_{1}}{C_{1}+C_{gs7}\left(1+g_{ds9}R_{1}\right)}$$

(4) $$C_{Eq}=C_{gs1}+\frac{C_{1}*C_{gs7}\left(1+g_{ds7}R_{1}\right)}{C_{1}+C_{gs7}\left(1+g_{ds9}R_{1}\right)}$$

(5) $$R_{Eq}=\frac{g_{ds7}}{\left(gm_{1}*gm_{7}\right)\left(gm_{5}*gm_{6}\right)}\left(1-\frac{\omega^{2}C_{1}C_{gs9}C_{gs7}R_{1}}{gm_{7}\left(gm_{9}+g_{ds9}\right)}\right)$$

(6) $$L_{Eq}=\frac{C_{gs1}}{gm_{7}*gm_{1}}\left(1-\frac{\omega^{2}\left(C_{gs6}C_{gs5}\right)}{gm_{5}*gm_{6}}\right)+\frac{C_{1}+C_{gs9}\left(1+g_{ds9}*R_{1}\right)}{gm_{7}\left(gm_{9}+g_{ds9}\right)}$$

(7) $$\omega_{0}=\frac{\sqrt{gm_{7}*\left(gm_{9}+g_{ds9}\right)}}{C_{1}C_{gs9}C_{gs7}R_{1}}$$

(8) $$Q(\omega_{0})=\frac{\omega_{0}L_{Eq}}{R_{Eq}}=\frac{{Gm}_{1}{Gm}_{2}}{g_{ds7}}\frac{\sqrt{gm_{7}C_{1}C_{gs9}R_{1}*\left(gm_{9}+g_{ds9}\right)}}{C_{gs7}}$$

where, $gm_{1}*gm_{7}={Gm}_{1}$ and $gm_{5}*gm_{6}={Gm}_{2}$ as it is reported in (8).

For $2gm_{1}+gm_{3}>g_{ds7}$, the gate voltage is used to modulate the drain conductance $g_{ds7}$, which is an efficient approach for fine-tuning the inductance. Numerous essential properties of a constructed active inductor can be used to provide a decent representation of the device’s efficiency. Such properties are dependent solely on the layout strategies adopted, and as a result, on the implicit electrical characteristics of active inductors, as stated in the above equations.

### 2.1. The Frequency Spectrum

The design of the inductor can also be modeled as a lossy gyrator-C. Then, it can be implied that it will exhibit inductive activity across a relatively restricted frequency band. For an inductor of such a class, it is critical to ensure that it will operate inductively over the required cellular radio frequency spectrum.

### 2.2. Tunability of the Inductor

For developing the frequency selective configurations, the tuning range is an imperative part of the active inductor. $g_{m1}$ and $g_{m2}$ are the values of transconductance that can be utilized to calibrate the inductor’s value.

### 2.3. The Q-Factor

Researchers must minimize significant losses in various applications, and notably in transmission systems. Inductors have a greater Q-factor which results in a decrease in losses. The quality factor values for the inductors of the passive CMOS are low, to clarify what we discussed in the introductory part. The objective of this design when constructing the active inductor is to gain a quality factor of above one thousand.

### 2.4. The Consumption

In contrast to passive inductors, active inductors help with the power consumption. Consumption by a typical gyrator-C inductor is determined by the L value of the device. A compact inductor is obtained by having large $g_{m1}$ and $g_{m2}$, whilst a large inductor is achieved by having low transconductances. Even though the consumption of gyrator-C is indeed not crucial, the use of small inductors still indicates a higher level of consumption than would otherwise be expected. With the addition of inbuilt linearization or Q enhancement circuits, consumption will become an essential characteristic of the active inductors configuration. This is discussed with the measurement results in further detail in the following section.

### 2.5. The Noise

By far, noise is the most significant disadvantage of CMOS active inductors in comparison with passive inductors. Mostly, during noise analysis, they display stronger $G_{ds2}$, gm1, and $C_{gs2}$, and $g_{m2}$ is more on the noise level. As for the constant $g_{m2}$, the influence of noise will be considerably larger for small inductors than for bigger ones. Whenever active inductors are being used in receiver functional units, often including LNAs, the distortion may be a key design attribute to take into consideration. In order to aim our analysis towards transmission systems, we outlined the noise benchmarks −112.43 at 1 MHz and −133.27 dBc/Hz at 10 MHz away from the carrier in GSM band, where the RFIC output power is near 12 dBm.

## 3. Proposed TAI-VCO Circuit Design

**Figure 7:**
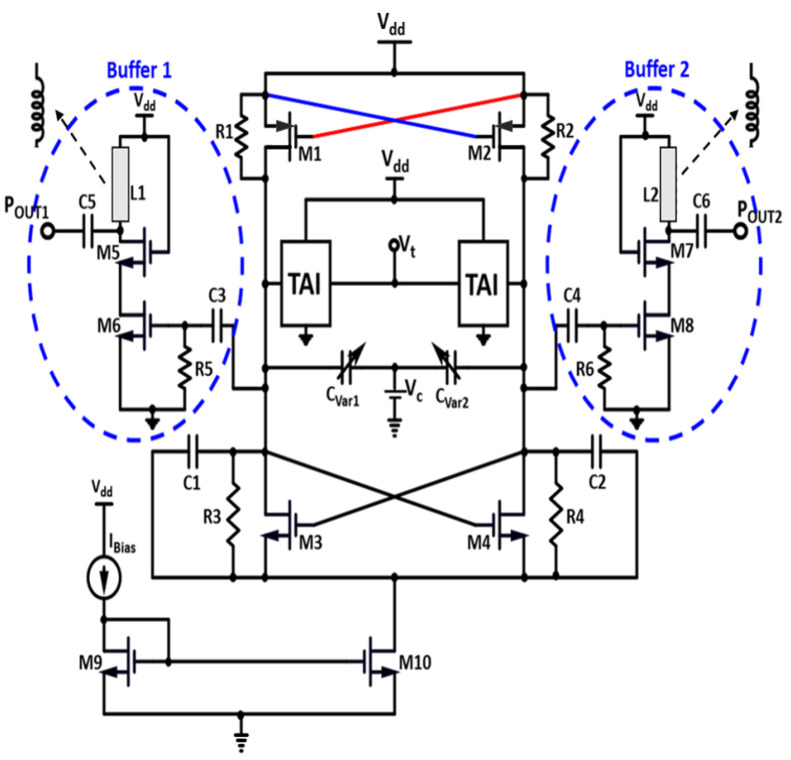
Schematic diagram of the proposed CTAI-VCO.

A theoretical representation of the advanced VCO is shown in Figure 7, wherein the negative conductance is employed in the LC tank to recompense for the LC tank loss, and the LC tank is comprised of two TAI circuits along with two varactors, $C_{Var1}$ and $C_{Var2}$, for the frequency control. This figure depicts a graphical illustration of the stated VCO with a low voltage and high Q-factor. AI facilitates the VCO’s performance in terms of both power consumption and phase noise. Though an active inductor’s equivalent inductance can be calibrated across a wide array of frequencies, it can be used as an implication to acquire coarse frequency tuning or selection of a band. Furthermore, in the LC tank, the varactor for fine-tuning is used, retaining relatively low sensitivity of tuning to ensure uniformity in frequencies. In addition, to boost the properties of the VCO, the TAI is deployed. The equivalent inductance of the AI is regulated by $V_{t}$. The constraints of the tuning range of the VCO can be eliminated by assessing the maximum to minimum inductance range used as a varactor for regulating the effective capacitance by $V_{c}$ in accumulation mode.

As a potential outcome, wideband service is carried out with continuous frequency tuning. MOS devices better can be configured for the appropriate tuning sensitivity, despite demeaning the VCO optimum tuning range, even though it is for fine-tuning only. M1–M2 PMOS cross-coupled transistors have connected R1 and R2. Concerning loss compensation, the M3–M4 NMOS cross-coupled pair deliver negative conductance. A current-reuse bias approach is presented to attenuate the consumption of power by assembling the cross-coupled pair with the cascode AI model. A higher amount of $V_{c}$ enables the equivalent inductance of the AI to rise, resulting in a reduction in the output frequency of the VCO, or vice versa. Transistors M7 and M8 are embedded to provide negative transconductance that reduces for all the losses of the LC tank. The M3 and M4 transistors are distorted by current mirrors M9 and M10, which at least for the signal, maintain the interface node isolated from the ground and permit a certain differential application. Two buffers, $P_{OUT1}$ (+ve node) and $P_{OUT2}$ (−ve node), are employed to drive the 50 ohms load, connected with $C_{3}$ and $C_{4}$, respectively, by neglecting the inductances $L_{1}$ and $L_{2}$.

**Figure 8:**
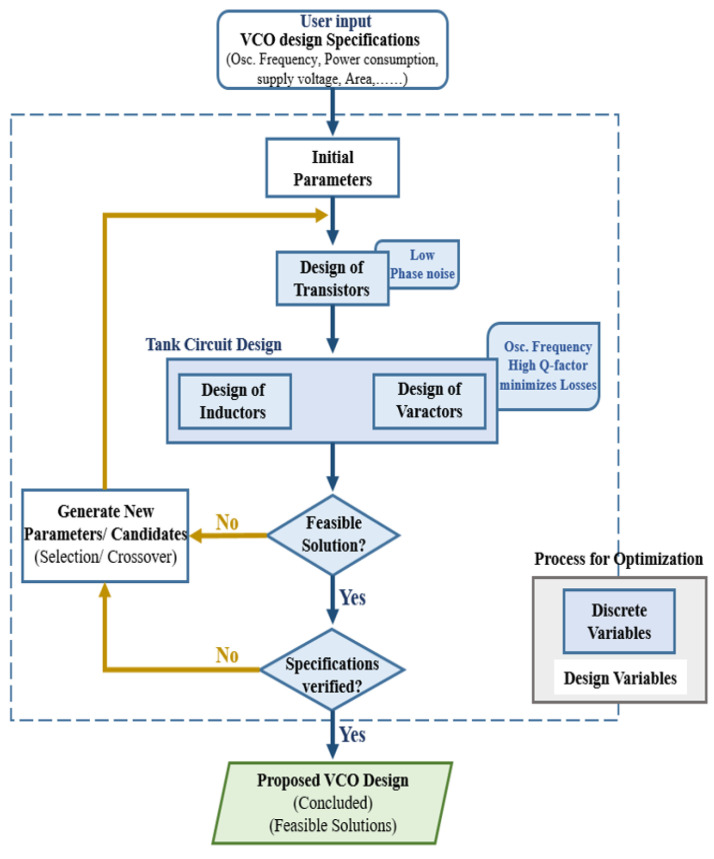
Designed VCO design flowchart.

Figure 8 is a design flow representation of the AI-based VCO technique. Acknowledging the TAI-VCO network model depicted in Figure 8, the first step in the design concept is the development of the AI, which is outlined in Figure 4. This is executed because the drain current in each transistor is the primary factor that contributes to the phase noise. The subsequent steps in the modeling process involve enhancing both the relevant Q and the tank tuning range to provide the excellent outcomes. As a result, the primary objective of the integrated design approach is to reduce the amount of phase noise and the amount of power that the system consumes.

Table 2 presents the design component values of the proposed VCO buffer, as drawn in Figure 7. Buffer 1 and 2, as shown in the above layout, contain equivalent values for the respective components executed in the circuit configuration and shown in this table.

## 4. Measurement Results and Discussion

The VCO is composed of cross-coupled amplifier, buffers, and a resonance circuit. $C_{Var1}$ and $C_{Var2}$ are variable capacitors controlled by controlled voltage $V_{c}$; and two cross-coupled circuit designs are implemented by the transistors $M_{1}$ and $M_{2}$ connected with the $V_{dd}$, and the transistors $M_{3}$ and $M_{4}$ connected to the current mirror circuit. A cross-coupled amplifier is used to offset the equivalent resistance of the resonant circuit shown in Figure 4. $g_{m}=g_{m1}=g_{m2}$, as M1 and M2 are transistors with the same parameters. The cross-coupled amplifier could generate a negative resistance: $R_{eq}=\frac{-2}{g_{m}}$. When the equivalent resistance of resonant circuit is $R_{p}$, for oscillation occurrence, the negative resistance must cancel the loss of the tank: $\frac{-2}{g_{m}}<R_{p}$, and hence $g_{m}R_{p}>2$. $g_{m}$ of the cross-coupled amplifier will decay with the increase in frequency due to the variable capacitors and parasitic capacitors. Therefore, the $g_{m}R_{p}$ must be larger in the various Si technology corners to ensure the startup of the oscillator. However, the parasitic capacitors connected to the tank must be considered when the VCO works at a higher frequency, as the parasitic capacitance of transistor may be comparable to or even larger than the varying capacitance.

**Figure 9:**
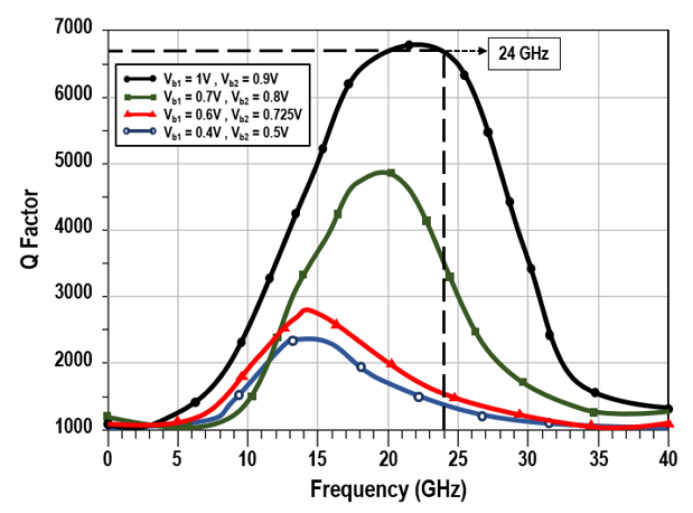
Quality factor of the designed TAI with varying bias voltage.

**Figure 10:**
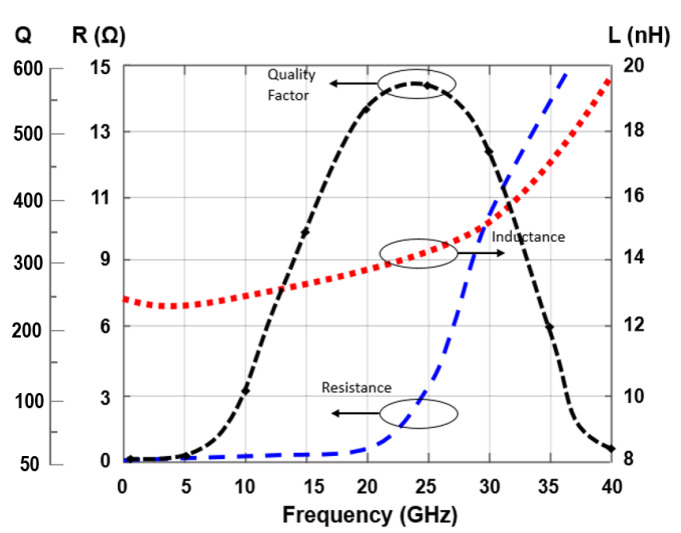
Quality factor of the designed VCO.

Figure 9 is an illustration of the variations in the Q factor for TAI resulting from changes in the bias voltages $V_{b1}$ and $V_{b2}$, while keeping $V_{dd}$ as 0.9 V in the circuit layout. A highest attainable quality factor of 6825 was calculated at a frequency of 24 GHz, while maintaining the bias voltages at $V_{b1}=1$ V and $V_{b2}=0.9$ V. The quality factor of the designed VCO is modulated by the inductance and resistance value of the circuit, which is gained by operating the advanced tunable active inductor. A higher quality factor can be achieved as explained in Figure 10 in a wide frequency tuning range, from 21.79 to 29.92 GHz. However, the Q-factor of value 546 is obtained at the 24 GHz frequency band of the proposed VCO, which is the key required parameter of this report for automotive radar applications.

### Experimental Results and Analysis

Furthermore, the declines in the conductance and parasitic resistance of the TAI as results of the $C_{ad}$ lead to a significant enhancement in the AI-VCO phase noise. Since $C_{ad}$ is accepted, there is a trade-off between the phase noise and the tuning range. A figure of merit value $\left(FOM_{T}\right)$, which along with the tuning range is used to determine the gradual efficiency of the VCO, is defined as [18];

(9) $$FoM_{T}=PN\left(\Delta f\right)-20log_{10}(\frac{f_{osc}}{\Delta f})+10log_{10}\left(P_{dc},mW\right)$$

**Figure 11:**
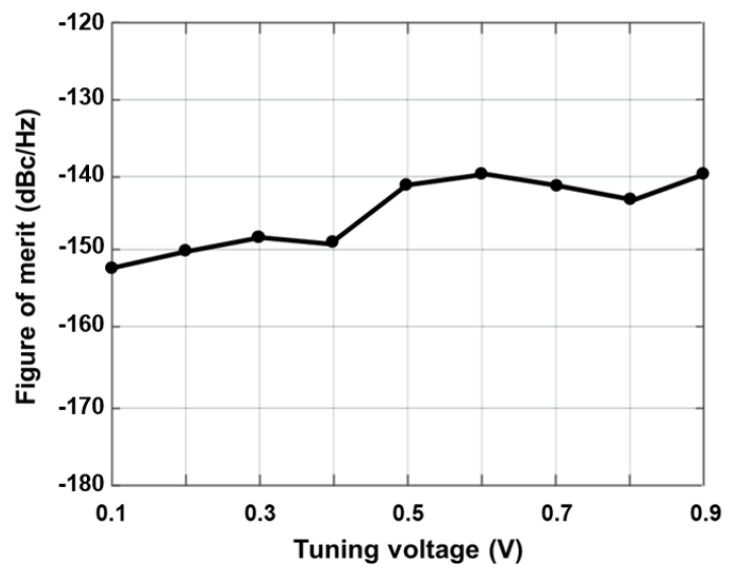
Figure of merit of the designed VCO.

**Figure 12:**
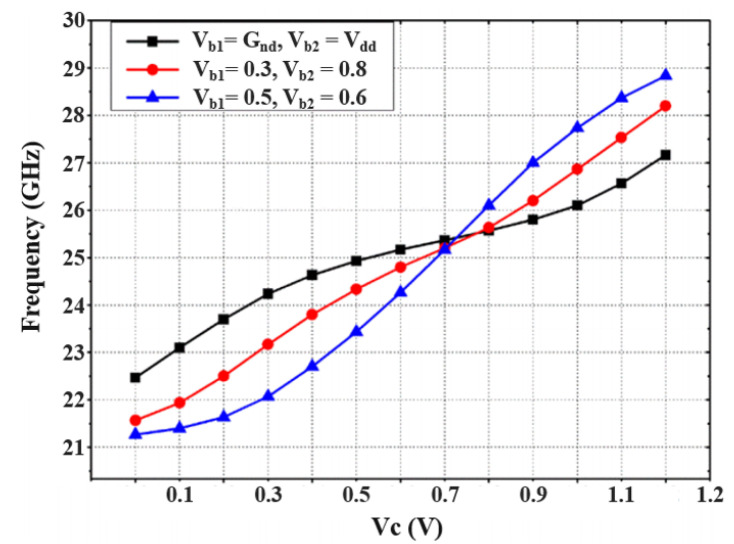
Frequency tuning graph with $V_{c}$ for different $V_{b1}$ and $V_{b2}$ parameters.

VCO attains an FoM of −153.48 dBc/Hz by considering the tuning voltage as reported in Figure 11. The resultant inductance values for this operating range are 6.9–16.2 nH, although the series resistance is maintained below 0.59 Ω throughout. Despite the fact that the additional capacitor $C_{ad}$ has minor negative effects on the tuning range frequency and frequency of oscillation, it has a beneficial impact on phase noise. By considering the biased voltages $V_{b1}$ and $V_{b2}$ at variable voltage parameters, along with the controlled voltage $V_{c}$, the frequency tuning graphs were drawn in Figure 12.

**Figure 13:**
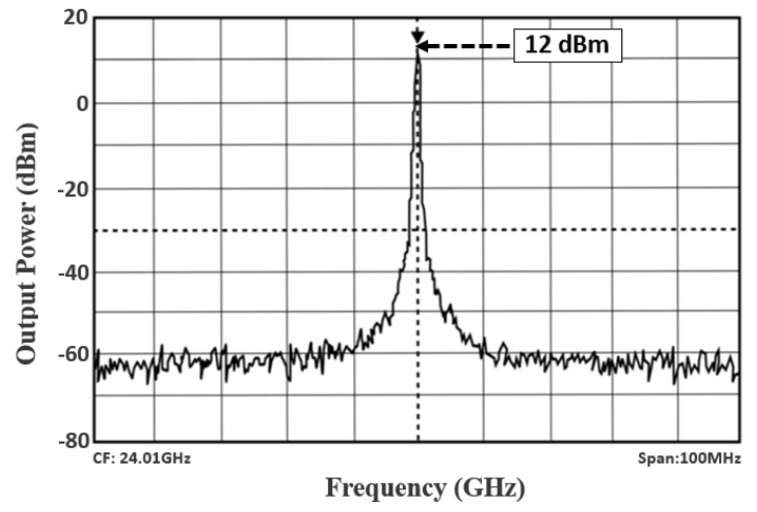
Measured output power spectrum of the VCO.

**Figure 14:**
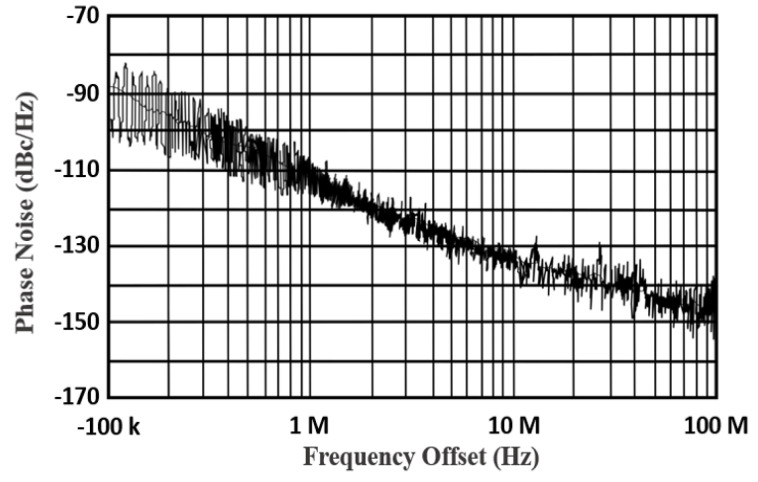
Measured phase noise of the VCO.

**Figure 15:**
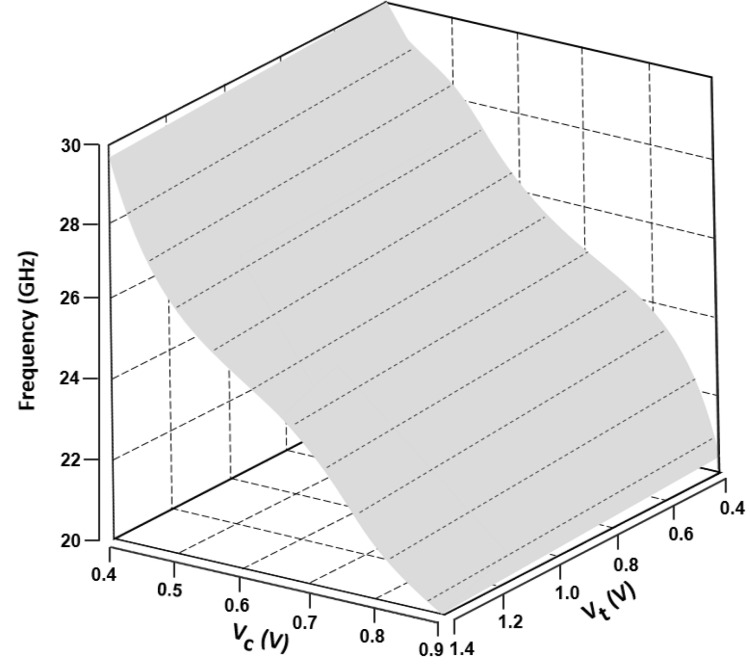
Graphical representation of the wide frequency tuning range of the VCO.

**Figure 16:**
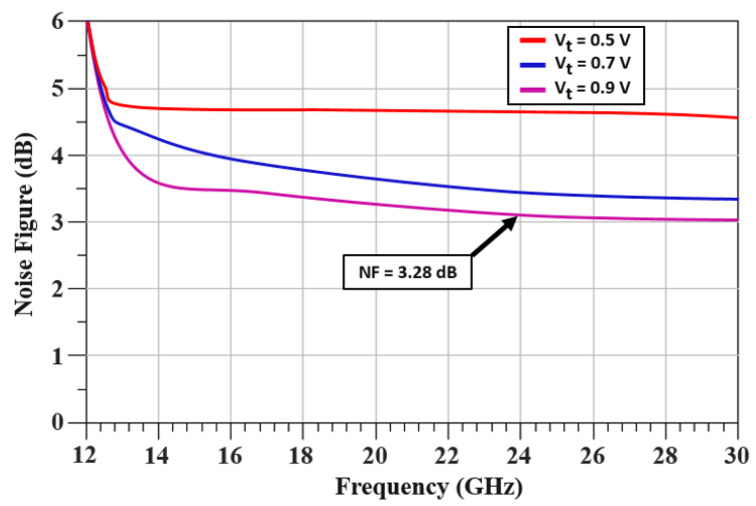
Noise figure for various tuning voltages.

Figure 13 displays the output power spectrum at the central frequency of 24.01 GHz: 12 dBm of maximum output power is delivered by the VCO. The measured phase noise performance of the proposed TAI-VCO at 1 MHz offset frequency is −112.43 or −133.27 dBc/Hz at 10 MHz, as shown in Figure 14. Figure 15 shows the graphical representations of the controlled voltage $V_{c}$ and the tuning voltage $V_{t}$, which span from 0.4 to 0.9 V and 0.4 to 1.4 V, respectively, over the fine frequency tuning range of the VCO. Noise of 3.28 dB was calculated for the required 24 GHz frequency spectrum applications while using 0.9 V as the operating voltage, as described in Figure 16. The FoM is a clear and intrinsic process of assessing various topologies of VCO. The phase noise responses to the frequency of oscillation and power consumption were normalized.

**Figure 17:**
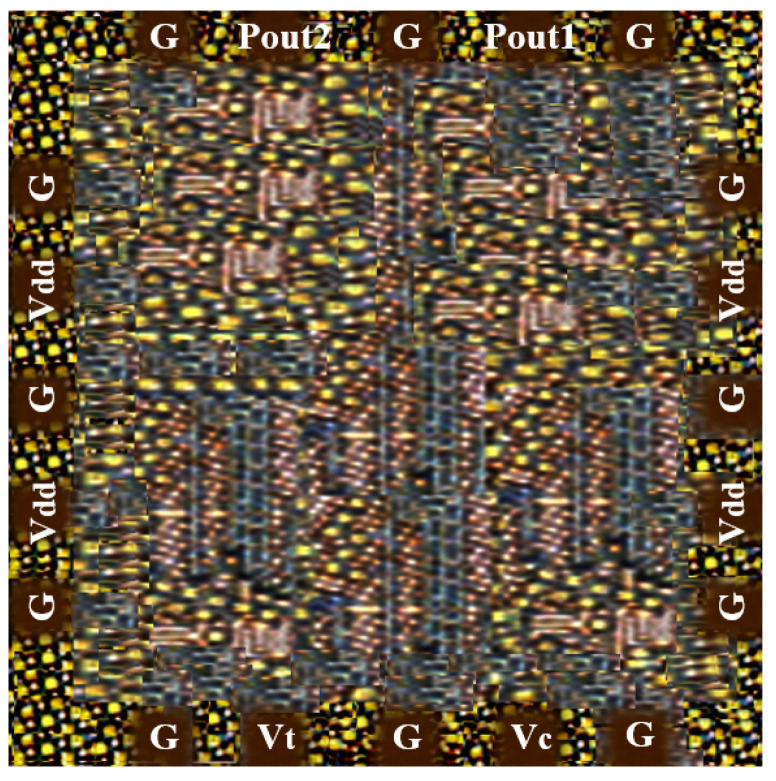
Layout of the proposed VCO.

**Figure 18:**
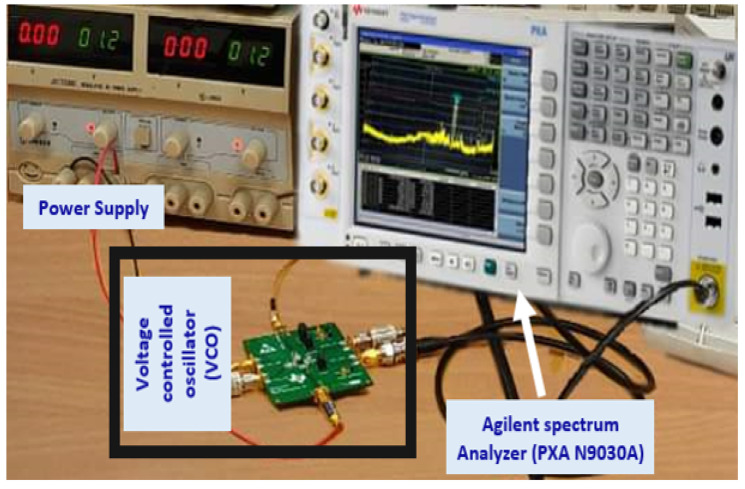
Experimental set-up of the proposed research.

The power consumption of the VCO ranges from 12.61 to 23.12 mW depending on the specified frequency tuning range. Advanced Design System (ADS) was adopted for the analytical simulation results and the complete circuit measurements, which were performed by using EM tools and cadence. The final layout of the novel VCO circuit design, which was made by using 65 nm CMOS technology, was implemented as displayed in Figure 17, and the chip area of the VCO was 0.142 × 0.031 mm${}^{2}$. The experimental set-up of the entire research was as shown in Figure 18. Table 3 illustrates the performance summary of the state-of-art compared to the wide-FTR VCO. Related analysis was carried out at the RF front-end, which clearly demonstrated complete system outcomes from time to time in compliance with the receiver requirements.

## 5. Conclusions and Future Works

A novel design of an active inductor-based VCO with variable output oscillation frequency has been introduced in this research article, which produces an oscillation frequency that varies from 22 to 29 GHz. It uses K-band and Ka-band frequency spectra and can be tuned to any desired frequency response. The simulation of the configuration was aimed at developing a tuning scope of 89%, and it has a DC power dissipation range of 12.61 to 23.12 mW. Some of the most important characteristics of this design concept are its high differential output power, good phase noise performance, high figure of merit, and small silicon area usage. The proposed design demonstrated a maximum Q-factor of AI 6825. The desirable phase noise variations of the proposed VCO are −112.43 and −133.27 dBc/Hz at 1 and 10 MHz offset frequencies for 24 GHz applications with a noise figure (NF) of 3.28 dB under a 0.9 V power supply.

## Figures and Tables

**Table 1:** Comparison study of the passive and active inductors.

Performances	Passive Inductor (Spiral)	Active Inductor (Simulated: Active Device)
**Q-factor**	Low quality factor	High quality factor
**Tunability**	Narrow	Wide
**Die-Area**	Needs broad space	Needs limited space
**Power Consumption**	Zero	Significant
**Linearity**	Good Linearity	Rough Linearity
**Noise**	Pleasant	Poor: poor phase noise performance
**EMI**	Compelling	EMI insensitive
**Floor-Planning**	Unacceptable	Not required

**Table 2:** The circuit component values of the proposed VCO-buffer.

Components	Values
$M_{7}$	23/65 nm
$M_{8}$	16.85/65 nm
$C_{6}$	170.27 fF
$C_{4}$	96 fF
$R_{6}$	11.52 Ω

**Table 3:** Performance summary of the state-of-art and the wide-FTR VCO.

Ref.	Topology	FTR [%]	PN [dBc/Hz]	Area [mm${}^{2}$]	FoM [dBc/Hz]	Power [mW]	FTR [GHz]	CMOS Process
[1]	AI	143	−101∼−118 @ 1 MHz	0.045	-	6∼28	0.5∼3.0	180 nm
[10]	CML	-	−104 @ 1 MHz	TX: 1.9 × 1.4 RX: 1.8 × 0.9	-	495	23.8∼24.5	130 nm
[13]	AI	120	−78∼−90 @ 1 MHz	0.09	-	13.8	0.5∼2.0	180 nm
[21]	AI	-	−80∼−93 @ 1 MHz	80 × 120 µm ${}^{2}$	-	16.27	0.1∼2.5	180 nm
[22]	Body bias PMOS Varactor	20.4	−138.4 @ 1 MHz	0.666	−201.2	13.1	4.5∼5.5	180 nm
[23]	AI	-	−92.2 @ 1 MHz	171 × 174 µm ${}^{2}$	−166	2.2∼13	1.13∼3.24	180 nm
[26]	Integer PLL	-	−104.32 @ 1 MHz −127.02 @ 10 MHz	0.776 × 0.726	1.37 (GHz/degree rms × mW)	3.52	24∼25.8	65 nm
[27]	-	78	−119∼−128 @ 10 MHz	0.02	−188∼−197	-	10.5∼24	32 nm
[28]	-	23	−101 @ 1 MHz	0.7	-	88	23.8∼26.4	130 nm
[29]	Quadrature	-	−110.5∼−104.2 @ 1 MHz	-	-	14.4	2.5	180 nm
[30]	BAC Architecture	53	−109.7 @ 1 MHz	0.12	−187.9∼−181.3	3.9∼5	11.3	65 nm
[31]	Triple-coupled Transformer	17	−111.9 @ 10 MHz	260 × 250 µm ${}^{2}$	−184	6.2	50.1∼59.8	65 nm
[32]	SSI	40.3	−119 @ 10 MHz	200 × 250	-	4.3	21∼31.6	65 nm
**This work**	**Active Inductor (AI)**	**86**	**−112.43 @** **1 MHz −133.27 @** **10 MHz**	**0.142 × 0.031**	**−153.48**	**23.12**	**21.79∼29.92**	**65 nm**

## Data Availability

Not applicable.

## References

[1] Lu L.H., Hsieh H.H., Liao Y.T. (2006). A wide tuning-range CMOS VCO with a differential tunable active inductor. IEEE Trans. Microw. Theory Tech..

[2] Manetakis K., Park S.M., Payne A., Setty S., Thanachayanont A., Toumazou C. Wideband CMOS analog cells for video and wireless communications. Proceedings of the IEE Colloquium Wideband Circuits, Modelling and Techniques.

[3] Yang Z., Chen Y., Yang S., Mak P.-I., Martins R.P. (2019). A 10.6-mW 26.4-GHz Dual-Loop Type-II Phase-Locked Loop Using Dynamic Frequency Detector and Phase Detector. IEEE Access.

[4] Lee D.-G., Nikoofard A., Mercier P.P. (2020). A 254.1-dB FoM 2.4-GHz Subsampling PLLWith a 76-dBc Reference Spur by Employing a Varactor-Based Cancellation Technique. IEEE Solid-State Circuits Lett..

[5] Kim B.-H., Hong Y., An Y.-J., Kim S.-G., Lee H.-J., Kim S.-W., Hong S.-B., Yun G.-H., Yook J.-G. (2016). A Proximity Coupling RF Sensor for Wrist Pulse Detection Based on Injection-Locked PLL. IEEE Trans. Microw. Theory Tech..

[6] Pimenta M., Gurleyuk C., Walsh P., O’Keeffe D., Babaie M., Makinwa K.A.A. (2021). A 200-µW Interface for High-Resolution Eddy-Current Displacement Sensors. IEEE J. Solid-State Circuits.

[7] Tang L., Wang Z., Qiu Y., Zhang C., Xu J. (2015). An ultra-high speed monolithic clock recovery circuit in 0.2-µm GaAs process. Analog. Integr. Circuits Signal Process.

[8] Drago S., Leenaerts D.M.W., Nauta B., Sebastiano F., Makinwa K.A.A., Breems L.J. (2010). A 200 µA Duty-Cycled PLL forWireless Sensor Nodes in 65 nm CMOS. IEEE Solid State Circuits.

[9] Avila H.E.D.L., de Andrade G.A., de Sousa F.R., Pagano D.J. (2019). Modeling and Analysis of a PLL-Based Resonant Frequency Tracking System Using a Resonant Cavity Sensor. IEEE Sens. J..

[10] Pyo G., Yang J., Ku H., Kim C.Y., Hong S. K-band FMCW radar CMOS front-end ICs with 13.3 dBm output power. Proceedings of the IEEE Radio Frequency Integrated Circuits Symposium.

[11] Gresham I., Jenkins A., Egri R., Eswarappa C., Kinayman N., Jain N., Anderson R., Kolak F., Wohlert R., Bawell S.P. (2004). Ultra-wideband radar sensors for short-range vehicular applications. IEEE Trans. Microw. Theory Tech..

[12] Kang C.W., Moon H., Yang J.R. (2021). Switched-Biasing Techniques for CMOS Voltage-Controlled Oscillator. Sensors.

[13] Mukhopadhyay R., Park Y., Sen P., Srirattana N., Lee J., Lee C.H., Nuttinck S., Joseph A., Cressler J.D., Laskar J. (2005). Reconfigurable RFICs in Si-based technologies for a compact intelligent RF front-end. IEEE Trans. Microw. Theory Tech..

[14] Hsiao C.C., Kuo C.W., Ho C.C., Chan Y.J. (2002). Improved quality-factor of 0.18- µm CMOS active inductor by a feedback resistance design. IEEE Microw. Wirel. Components Lett..

[15] Burghartz J.N., Jenkins K.A., Soyuer M. (1996). Multilevel-spiral inductors using VLSI interconnect technology. IEEE Electron Device Lett..

[16] Park M., Lee S., Yu H.K., Koo J.G., Nam K.S. (1997). High Q CMOS compatible microwave inductors using double-metal interconnection silicon technology. IEEE Microw. Guid. Wave Lett..

[17] Chen P.Q., Chan Y.J. (2000). Improved microwave performance on low-resistivity Si substrates by Si ion implantation. IEEE Trans. Microw. Theory Tech..

[18] Hara S., Tokumitsu T., Tanaka T., Aikawa M. (1988). Broad band monolithic microwave active inductor and its application to miniaturise wide band amplifiers. IEEE Trans. Microw. Theory Tech..

[19] El Khoury S.G. (1996). New approach to the design of active floating inductors in MMIC technology. IEEE Trans. Microw. Theory Tech..

[20] Pascht A., Fischer J., Beeroth M. A CMOS low noise amplifier at 2.4 GHz with active inductor load. Proceedings of the 2001 Topical Meeting on Silicon Monolithic Integrated Circuits in RF Systems. Digest of Papers (IEEE Cat. No. 01EX496).

[21] Babaei Kia H., Khari A’ain A., Grout I. (2014). Wide tuning-range CMOS VCO based on a tunable active inductor. Int. J. Electron..

[22] Lu K.C., Wang F.K., Horng T.S. (2013). Ultralow phase noise and wideband CMOS VCO using symmetrical body-bias PMOS varactors. IEEE Microw. Wirel. Components Lett..

[23] Basaligheh A., Saffari P., Winkler W., Moez K. (2019). A wide tuning range, low phase noise, and area efficient dual-band millimeter-wave CMOS VCO based on switching cores. IEEE Trans. Circuits Syst. I Regul. Pap..

[24] Jeong Y.J., Kim Y.M., Chang H.J., Yun T.Y. (2012). Low-power CMOS VCO with a low-current, high-Q active inductor. IET Microw. Antennas Propag..

[25] Momen H.G., Yazgi M., Kopru R., Saatlo A. A new high performance CMOS active inductor. Proceedings of the 39th International Conference on Telecommunications and Signal Processing (TSP).

[26] Siddique A., Ryu J.Y. (2020). A 24 GHz frequency synthesizer for automotive collision avoidance radar. Int. J. Electron. Lett..

[27] Sadhu B., Ferriss M., Friedman D. A capacitance boosted full-octave LC VCO based 0.7 to 24 GHz fractional-N synthesizer. Proceedings of the IEEE Radio Frequency Integrated Circuits Symposium (RFIC).

[28] Issakov V., Tiebout M., Mertens K., Cao Y., Thiede A., Simbürger W., Maurer L. A compact low-power 24 GHz transceiver for radar applications in 0.13 µm CMOS. Proceedings of the IEEE International Conference on Microwaves, Communications, Antennas and Electronics Systems.

[29] Lu C.T., Hsieh H.H., Lu L.H. (2009). A low-power quadrature VCO and its application to a 0.6-V 2.4-GHz PLL. IEEE Trans. Circuits Syst. I Regul. Pap..

[30] Agarwal P., Chahardori M., Heo D. (2020). A new boosted active-capacitor with negative-G m for wide Tuning range VCOs. IEEE Trans. Circuits Syst. I Regul. Pap..

[31] Kashani M.H., Tarkeshdouz A., Molavi R., Sheikholeslami A., Afshari E., Mirabbasi S. (2019). On the design of a high-performance mm-wave VCO with switchable triple-coupled transformer. IEEE Trans. Microw. Theory Tech..

[32] Agarwal P., Sah S.P., Molavi R., Mirabbasi S., Pande P.P., Oh S.E., Kim J.H., Heo D. (2017). Switched substrate-shield-based low-loss CMOS inductors for wide tuning range VCOs. IEEE Trans. Microw. Theory Tech..

